# Supplementary material for: Correlation induced electron-hole asymmetry in quasi- two-dimensional iridates
Source: Nat Commun. 2017 Sep 25;8:686. doi: 10.1038/s41467-017-00818-8 (PMC5612937; doi:10.1038/s41467-017-00818-8)
Supplement: Supplementary file 1 — Supplementary Information [file 41467_2017_818_MOESM1_ESM.pdf]

Description of Supplementary Files

File Name: Supplementary Information
Description: Supplementary Figure, Supplementary Notes and Supplementary References

File Name: Peer Review File
Description:

SUPPLEMENTARY NOTE 1:
MAGNON DISPERSION – DETAILED FORM OF $\mathcal{H}_{\mathrm{mag}}$

The interaction between the $j = 1/2$ isospins in the quasi-two-dimensional iridates is well described by the Heisenberg Hamiltonian [1]. Using the successive Holstein-Primakoff, Fourier, Bogoliubov transformations and skipping the terms describing the (iso)magnon interactions, we obtain the Heisenberg Hamiltonian in the usual "linear spin-wave approximation" form [2]:

$$\mathcal{H}_{\mathrm{mag}} = \sum_{\mathbf{q}} \omega_{\mathbf{k}} (\alpha^{\dagger}_{\mathbf{q}} \alpha_{\mathbf{q}} + \beta^{\dagger}_{\mathbf{q}} \beta_{\mathbf{k}}), \tag{1}$$

where $|\alpha_{\mathbf{q}}\rangle$ and $|\beta_{\mathbf{q}}\rangle$ are the single magnon states, $\mathbf{q}$ is the crystal momentum and $\omega_{\mathbf{q}}$ is the magnon dispersion relation given by

$$\omega_{\mathbf{q}} = \sqrt{A^2_{\mathbf{q}} - B^2_{\mathbf{q}}} \tag{2}$$

with

$$A_{\mathbf{q}} = 2(J_1 - J_2 + J_2 \cos q_x \cos q_y - J_3(1 - \frac{1}{2}(\cos 2q_x + \cos 2q_y))), \tag{3}$$

$$B_{\mathbf{q}} = J_1(\cos q_x + \cos q_y). \tag{4}$$

Here J_1, J_2 and J_3 are the nearest, next nearest and third neighbor isospin exchange interactions, respectively.

We note at this point that the the parameters of the Bogoliubov transformation, the so-called Bogoliubov coefficients $u_{\mathbf{q}}$, $v_{\mathbf{q}}$, are given by the following, well-known, expressions in the linear spin-wave theory:

$$\begin{aligned} u_{\mathbf{q}} &= \frac{1}{\sqrt{2}} \sqrt{\frac{A_{\mathbf{q}}}{\omega_{\mathbf{q}}} + 1}, \\ v_{\mathbf{q}} &= -\frac{\mathrm{sign}(B_{\mathbf{q}})}{\sqrt{2}} \sqrt{\frac{A_{\mathbf{q}}}{\omega_{\mathbf{q}}} - 1} \end{aligned} \tag{5}$$

where the coefficients $A_{\mathbf{q}}$ and $B_{\mathbf{q}}$ are defined above.

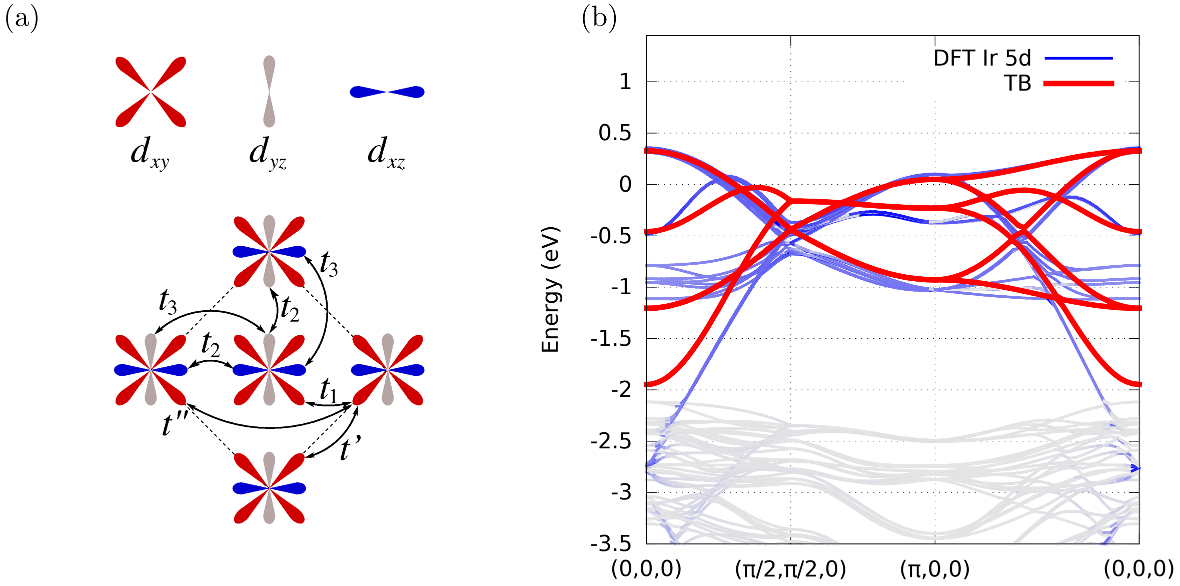

Supplementary Figure 1. **Tight-binding model** (a) A cartoon illustrating the t_1, t_2, t_3, t' and t'' hopping paths between the Ir-5d-t_{2g} orbitals taken into account in the tight-binding model. (b) Comparison between the density functional theory (DFT) band structure as calculated for Sr_2IrO_4 (blue and grey lines) and the tight-binding (TB) model written in the Ir-5d-t_{2g} orbital basis (red lines). The intensity of the blue shade of the DFT bands represents the amount of the Ir-5d-t_{2g} character in a given band at a particular momentum (darkest blue – largest overlap with the Ir-5d-t_{2g} orbitals; grey – lowest overlap with the Ir-5d-t_{2g} orbitals). The Fermi energy is set to zero.

SUPPLEMENTARY NOTE 2: DETERMINING THE TIGHT-BINDING HAMILTONIAN FROM THE DFT CALCULATIONS

The electronic band-structure of Sr_2IrO_4 was calculated using DFT in the local density approximation [3] and within the linearized augmented plane wave approach using the WIEN2k code [4]. We considered the 10 K crystal structure of Sr_2IrO_4, with the space group $I41/acd$, as reported in Ref. [5]. The calculated band-structure of Sr_2IrO_4 is shown in Supplementary Figure 1 (a) along a path in the Brillouin zone of the $I41/acd$ unit cell. The bands with the predominant Ir-5d-t_{2g} character are highlighted in blue.

We used the calculated dispersion of the Ir-5d-t_{2g} bands to parameterize our tight-binding

(TB) model:

$$\mathcal{H}_{\rm TB} = -\, t_1 \sum_{\langle \mathbf{i},\mathbf{j}\rangle || \hat{x},\hat{y},\sigma} c^{\dagger}_{\mathbf{i}\sigma} c_{\mathbf{j}\sigma} - t_2 \sum_{\langle \mathbf{i},\mathbf{j}\rangle || \hat{y},\sigma} a^{\dagger}_{\mathbf{i}\sigma} a_{\mathbf{j}\sigma} - t_2 \sum_{\langle \mathbf{i},\mathbf{j}\rangle || \hat{x},\sigma} b^{\dagger}_{\mathbf{i}\sigma} b_{\mathbf{j}\sigma} - t_3 \sum_{\langle \mathbf{i},\mathbf{j}\rangle || \hat{y},\sigma} b^{\dagger}_{\mathbf{i}\sigma} b_{\mathbf{j}\sigma}$$
$$- \, t_3 \sum_{\langle \mathbf{i},\mathbf{j}\rangle || \hat{x},\sigma} a^{\dagger}_{\mathbf{i}\sigma} a_{\mathbf{j}\sigma} - t' \sum_{\langle\langle \mathbf{i},\mathbf{j}\rangle\rangle || \hat{x'},\hat{y'},\sigma} c^{\dagger}_{\mathbf{i}\sigma} c_{\mathbf{j}\sigma} - t'' \sum_{\langle\langle\langle \mathbf{i},\mathbf{j}\rangle\rangle\rangle || \hat{x''},\hat{y''},\sigma} c^{\dagger}_{\mathbf{i}\sigma} c_{\mathbf{j}\sigma} + h.c., \qquad (6)$$

where $a^{\dagger}$, $b^{\dagger}$, and $c^{\dagger}$ operator create an electron in the $d_{\rm yz}$, $d_{\rm xz}$, $d_{\rm xy}$ orbitals (respectively) with spin $\sigma = \pm\frac{1}{2}$, $\hat{x}$ and $\hat{y}$ indicate the directions of the nearest neighbor bonds in the xy plane of the quasi-two-dimensional iridate, and $\hat{x'} = \hat{x} - \hat{y}$ and $\hat{y'} = \hat{x} + \hat{y}$ ($\hat{x''} = 2\hat{x}$ and $\hat{y'} = 2\hat{y}$) indicate the directions of the next nearest (third) neighbor bonds in the xy plane of the quasi-two-dimensional iridate. The TB model includes the nearest neighbor hopping integrals t_1, t_2 and t_3 as well as the next nearest and third neighbor integral t' and t'' between the Ir-5d-$t_{2\rm g}$ orbitals, with their meaning explained in Supplementary Figure 1 (a).

We found that the following parameter values are both physically reasonable and give a satisfactory match between the DFT and TB model bands:$t_1 = -0.2239$ eV, $t_2 = -0.373$ eV, $t' = -0.1154$ eV, $t_3 = -0.0592$ eV,$t'' = -0.0595$ eV. The TB model band-structure based on these parameter values is shown in red in Supplementary Figure 1 (b). Let us also note that the generic structure of the TB Hamiltonian follows from the well-known symmetries of an effective TB Hamiltonian for the transition metal oxide with the $t_{2\rm g}$ orbital degrees of freedom: the electrons located in the $d_{\rm ab}$ orbital can solely hop in the ab plane.

SUPPLEMENTARY NOTE 3:
MOTION OF THE "5d^6 DOUBLON" – DETAILED FORM OF $\mathcal{H}_t^{\rm d}$

Having obtained the TB Hamiltonian we are now ready to derive the Hamiltonian which would describe the motion of the "5d^6 doublon" added to the Mott insulating ground state formed by the 5d^5 iridium ions of the (undoped) quasi-two-dimensional iridates due to the nonzero hopping elements of the TB Hamiltonian. This means that the main task here is to calculate the following matrix elements of the tight-binding Hamiltonian [Supplementary Equation (6) above] $\langle 5d^6_{\mathbf{i}} 5d^5_{\mathbf{j}} | \mathcal{H}_{\rm TB} | 5d^5_{\mathbf{i}} 5d^6_{\mathbf{j}} \rangle$. This is done in several steps:

Firstly, we calculate the above matrix elements in the appropriate eigenstates of ionic Hamiltonian of the 5d^5 and 5d^6 configurations (these states are listed in Fig. 1. of the main text). We note that these matrix elements do not explicitly depend on the strong

on-site spin-orbit coupling λ, though the form of the appropriate eigenstates of the ionic Hamiltonian (Fig. 1 of the main text) is of course due to the onset of strong on-site spin-orbit coupling λ. Secondly, we assume the so-called no double occupancy constraint, which follows from the implicitly assumed here limit of strong on-site Coulomb repulsion – which prohibits the creation of "unnecessary" "$5d^6$ doublons" once the electron added to the quasi-two-dimensional iridate $5d^5$ ground state hops between sites. Technically this amounts to the introduction of the projection operator which takes care of this constraint. Finally, following the path described for example in Refs. [6, 7] and introducing the slave-fermion formalism followed by Fourier and Bogoliubov transformations, we arrive at the following polaronic Hamiltonian which describes the motion of the "$5d^6$ doublon":

$$\mathcal{H}_t^{\mathrm{d}} = \sum_{\mathbf{k}} V_{\mathbf{k}}^0 \left(d_{\mathbf{k}\mathrm{A}}^\dagger d_{\mathbf{k}\mathrm{A}} + d_{\mathbf{k}\mathrm{B}}^\dagger d_{\mathbf{k}\mathrm{B}} \right) + \sum_{\mathbf{k},\mathbf{q}} V_{\mathbf{k},\mathbf{q}} \left(d_{\mathbf{k}-\mathbf{q}\mathrm{B}}^\dagger d_{\mathbf{k}\mathrm{A}} \alpha_{\mathbf{q}}^\dagger + d_{\mathbf{k}-\mathbf{q}\mathrm{A}}^\dagger d_{\mathbf{k}\mathrm{B}} \beta_{\mathbf{q}}^\dagger + h.c. \right), \quad (7)$$

with the free next-nearest and third- neighbor hopping

$$V_{\mathbf{k}}^0 = -\frac{4t'}{3}\gamma'_{\mathbf{k}} - \frac{4t''}{3}\gamma''_{\mathbf{k}}, \quad (8)$$

and the vertex

$$V_{\mathbf{k},\mathbf{q}} = -\frac{8(t_1+t_2+t_3)}{3\sqrt{2N}} \left(\gamma_{\mathbf{k}-\mathbf{q}} u_{\mathbf{q}} + \gamma_{\mathbf{k}} v_{\mathbf{q}} \right), \quad (9)$$

where $\gamma_{\mathbf{k}} = 1/2(\cos k_x + \cos k_y)$, $\gamma'_{\mathbf{k}} = \cos k_x \cos k_y$ and $\gamma''_{\mathbf{k}} = 1/2(\cos 2k_x + \cos 2k_y)$, N is the number of sites and the Bogoliubov coefficients $u_{\mathbf{q}}$ and $v_{\mathbf{q}}$ are given in Supplementary Note 1.

SUPPLEMENTARY NOTE 4:
MOTION OF THE "$5d^4$ HOLE" – DETAILED FORM OF $\mathcal{H}_t^{\mathrm{h}}$

The Hamiltonian which describes the motion of the "$5d^4$ hole" ($\mathcal{H}_t^{\mathrm{h}}$) is derived in a similar way as in the "$5d^6$ doublon" case described in Supplementary Note 3. However, due to the multiplet structure of the eigenstates of the ionic Hamiltonian of the $5d^4$ configuration (see Fig. 1 of the main text), its form is far more complex – in the low energy limit it describes the hopping of the four distinct eigenstates that can be formed by the "$5d^4$ hole" (singlet S, and three triplets T_σ; see main text):

$$\mathcal{H}_t^{\mathbf{h}} = \sum_{\mathbf{k}} \left(\mathbf{h}_{\mathbf{k}\mathrm{A}}^\dagger \hat{V}_{\mathbf{k}}^0 \mathbf{h}_{\mathbf{k}\mathrm{A}} + \mathbf{h}_{\mathbf{k}\mathrm{B}}^\dagger \hat{V}_{\mathbf{k}}^0 \mathbf{h}_{\mathbf{k}\mathrm{B}} \right) + \sum_{\mathbf{k},\mathbf{q}} \left(\mathbf{h}_{\mathbf{k}-\mathbf{q}\mathrm{B}}^\dagger \hat{V}_{\mathbf{k},\mathbf{q}}^{\alpha} \mathbf{h}_{\mathbf{k}\mathrm{B}} \alpha_{\mathbf{q}}^\dagger + \mathbf{h}_{\mathbf{k}-\mathbf{q}\mathrm{A}}^\dagger \hat{V}_{\mathbf{k},\mathbf{q}}^{\beta} \mathbf{h}_{\mathbf{k}\mathrm{B}} \beta_{\mathbf{q}}^\dagger + h.c. \right). \quad (10)$$

Here the free hopping matrix is

$$\hat{V}^{0}_{\mathbf{k}} = \begin{pmatrix} F_1 & 0 & -F_2 & 0 & 0 & P_2 & 0 & -P_1 \\ 0 & F_4 & 0 & 0 & P_1 & 0 & Q_1 & 0 \\ -F_2 & 0 & F_3 & 0 & 0 & Q_2 & 0 & Q_1 \\ 0 & 0 & 0 & 0 & -P_2 & 0 & Q_2 & 0 \\ 0 & P_1 & 0 & -P_2 & F_1 & 0 & F_2 & 0 \\ P_2 & 0 & Q_2 & 0 & 0 & 0 & 0 & 0 \\ 0 & Q_1 & 0 & Q_2 & F_2 & 0 & F_3 & 0 \\ -P_1 & 0 & Q_1 & 0 & 0 & 0 & 0 & F_4 \end{pmatrix}, \tag{11}$$

while the matrices containing vertices are

$$\hat{V}^{\alpha}_{\mathbf{k},\mathbf{q}} = \begin{pmatrix} 0 & L_3 & 0 & -L_3 & Y_1 & 0 & -W_2 & 0 \\ L_3 & 0 & L_1 & 0 & 0 & Y_4 & 0 & W_1 \\ 0 & L_1 & 0 & L_1 & -W_2 & 0 & Y_2 & 0 \\ -L_3 & 0 & L_1 & 0 & 0 & W_1 & 0 & Y_3 \\ 0 & 0 & 0 & 0 & 0 & L_4 & 0 & -L_4 \\ 0 & 0 & 0 & 0 & L_4 & 0 & L_2 & 0 \\ 0 & 0 & 0 & 0 & 0 & L_2 & 0 & L_2 \\ 0 & 0 & 0 & 0 & -L_4 & 0 & L_2 & 0 \end{pmatrix}, \tag{12}$$

$$\hat{V}^{\beta}_{\mathbf{k},\mathbf{q}} = \begin{pmatrix} 0 & L_4 & 0 & -L_4 & 0 & 0 & 0 & 0 \\ L_4 & 0 & L_2 & 0 & 0 & 0 & 0 & 0 \\ 0 & L_2 & 0 & L_2 & 0 & 0 & 0 & 0 \\ -L_4 & 0 & L_2 & 0 & 0 & 0 & 0 & 0 \\ Y_1 & 0 & W_2 & 0 & 0 & L_3 & 0 & -L_3 \\ 0 & Y_3 & 0 & W_1 & L_3 & 0 & L_1 & 0 \\ W_2 & 0 & Y_2 & 0 & 0 & L_1 & 0 & L_1 \\ 0 & W_1 & 0 & Y_4 & -L_3 & 0 & L_1 & 0 \end{pmatrix}, \tag{13}$$

The nearest neighbor free hopping $P(\mathbf{k})$, $Q(\mathbf{k})$ and the polaronic diagonal $Y(\mathbf{k},\mathbf{q})$ and

non-diagonal $W(\mathbf{k},\mathbf{q})$ vertex elements

$$P_1(\mathbf{k}) = \frac{2\left(2t_1 - t_2\right)}{3\sqrt{3}}\gamma_{\mathbf{k}} - \frac{2t_3}{3\sqrt{3}}\gamma_{\mathbf{k}}, \tag{14}$$

$$P_2(\mathbf{k}) = \frac{2t_2}{\sqrt{3}}\tilde{\gamma}_{\mathbf{k}} - \frac{2t_3}{\sqrt{3}}\tilde{\gamma}_{\mathbf{k}}, \tag{15}$$

$$Q_1(\mathbf{k}) = \frac{(4t_1 + t_2)}{3\sqrt{2}}\gamma_{\mathbf{k}} + \frac{t_3}{3\sqrt{2}}\gamma_{\mathbf{k}}, \tag{16}$$

$$Q_2(\mathbf{k}) = \frac{t_2}{\sqrt{2}}\tilde{\gamma}_{\mathbf{k}} - \frac{t_3}{\sqrt{2}}\tilde{\gamma}_{\mathbf{k}}, \tag{17}$$

$$W_1(\mathbf{k},\mathbf{q}) = \frac{t_3 - t_2}{\sqrt{2N}}\left(\tilde{\gamma}_{\mathbf{k}-\mathbf{q}}u_{\mathbf{q}} + \tilde{\gamma}_{\mathbf{k}}v_{\mathbf{q}}\right), \tag{18}$$

$$W_2(\mathbf{k},\mathbf{q}) = -\frac{4\left(2t_1 - t_2 - t_3\right)}{3\sqrt{3N}}\left(\gamma_{\mathbf{k}-\mathbf{q}}u_{\mathbf{q}} - \gamma_{\mathbf{k}}v_{\mathbf{q}}\right), \tag{19}$$

$$Y_1(\mathbf{k},\mathbf{q}) = -\frac{16\left(t_1 + t_2 + t_3\right)}{9\sqrt{2N}}\left(\gamma_{\mathbf{k}-\mathbf{q}}u_{\mathbf{q}} + \gamma_{\mathbf{k}}v_{\mathbf{q}}\right), \tag{20}$$

$$Y_2(\mathbf{k},\mathbf{q}) = -\frac{2\left(4t_1 + t_2 + t_3\right)}{3\sqrt{2N}}\left(\gamma_{\mathbf{k}-\mathbf{q}}u_{\mathbf{q}} + \gamma_{\mathbf{k}}v_{\mathbf{q}}\right), \tag{21}$$

$$Y_3(\mathbf{k},\mathbf{q}) = -\frac{4t_1 + t_2 + t_3}{3\sqrt{2N}}\gamma_{\mathbf{k}}v_{\mathbf{q}} - \frac{3\left(t_2 + t_3\right)}{\sqrt{2N}}\gamma_{\mathbf{k}-\mathbf{q}}u_{\mathbf{q}}, \tag{22}$$

$$Y_4(\mathbf{k},\mathbf{q}) = -\frac{4t_1 + t_2 + t_3}{3\sqrt{2N}}\gamma_{\mathbf{k}-\mathbf{q}}u_{\mathbf{q}} - \frac{3\left(t_2 + t_3\right)}{\sqrt{2N}}\gamma_{\mathbf{k}}v_{\mathbf{q}}, \tag{23}$$

with $\tilde{\gamma}_{\mathbf{k}} = 1/2(\cos k_x - \cos k_y)$;

The free hopping elements arising from the next-nearest and third neighbor hoppings

$$F_1(\mathbf{k}) = -\frac{4t'\gamma'_{\mathbf{k}}}{9} - \frac{4t''\gamma''_{\mathbf{k}}}{9}, \tag{24}$$

$$F_2(\mathbf{k}) = -\frac{8t'\gamma'_{\mathbf{k}}}{3\sqrt{6}} - \frac{8t''\gamma''_{\mathbf{k}}}{3\sqrt{6}}, \tag{25}$$

$$F_3(\mathbf{k}) = -\frac{2t'\gamma'_{\mathbf{k}}}{3} - \frac{2t''\gamma''_{\mathbf{k}}}{3}, \tag{26}$$

$$F_4(\mathbf{k}) = -\frac{t'\gamma'_{\mathbf{k}}}{3} - \frac{t''\gamma''_{\mathbf{k}}}{3}; \tag{27}$$

The polaronic next-nearest and third neighbor hopping elements

$$L_1(\mathbf{k},\mathbf{q}) = \frac{4t'}{3\sqrt{N}}\gamma'_{\mathbf{k}-\mathbf{q}}u_{\mathbf{q}} + \frac{4t''}{3\sqrt{N}}\gamma''_{\mathbf{k}-\mathbf{q}}u_{\mathbf{q}}, \tag{28}$$

$$L_2(\mathbf{k},\mathbf{q}) = \frac{4t'}{3\sqrt{N}}\gamma'_{\mathbf{k}}v_{\mathbf{q}} + \frac{4t''}{3\sqrt{N}}\gamma''_{\mathbf{k}}v_{\mathbf{q}}, \tag{29}$$

$$L_3(\mathbf{k},\mathbf{q}) = \frac{8t'}{3\sqrt{6N}}\gamma'_{\mathbf{k}-\mathbf{q}}u_{\mathbf{q}} + \frac{8t''}{3\sqrt{6N}}\gamma''_{\mathbf{k}-\mathbf{q}}u_{\mathbf{q}}, \tag{30}$$

$$L_4(\mathbf{k},\mathbf{q}) = \frac{8t'}{3\sqrt{6N}}\gamma'_{\mathbf{k}}v_{\mathbf{q}} + \frac{8t''}{3\sqrt{6N}}\gamma''_{\mathbf{k}}v_{\mathbf{q}}. \tag{31}$$

SUPPLEMENTARY REFERENCES

[1] Kim, B. H., Khaliullin, G. & Min, B. I. Magnetic Couplings, Optical Spectra, and Spin-Orbit Exciton in 5d Electron Mott Insulator Sr_2IrO_4. *Phys. Rev. Lett.* **109**, 167205 (2012).

[2] Kim, J. *et al.* Excitonic quasiparticles in a spin-orbit Mott insulator. *Nat. Commun.* **5**, 4453 (2014).

[3] Perdew, J. P. & Wang, Y. Accurate and simple analytic representation of the electron-gas correlation energy. *Phys. Rev. B* **45**, 13244–13249 (1992).

[4] Blaha, P., Schwarz, K., Madsen, G. K. H., Kvasnicka, D. & Luitz, J. *WIEN2K, An Augmented Plane Wave + Local Orbitals Program for Calculating Crystal Properties* (Karlheinz Schwarz, Techn. Universität Wien, Austria, 2001).

[5] Huang, Q. *et al.* Neutron Powder Diffraction Study of the Crystal Structures of Sr_2RuO_4 and Sr_2IrO_4 at Room Temperature and at 10K. *Journal of Solid State Chemistry* **112**, 355–361 (1994).

[6] Martinez, G. & Horsch, P. Spin polarons in the t-J model. *Phys. Rev. B* **44**, 317–331 (1991).

[7] Plotnikova, E. M., Daghofer, M., van den Brink, J. & Wohlfeld, K. Jahn-Teller Effect in Systems with Strong On-Site Spin-Orbit Coupling. *Phys. Rev. Lett.* **116**, 106401 (2016).
